# Neonatal Umbilical Cord Hematoma at the Fetal Insertion With Benign Clinical Course: A Case Report

**DOI:** 10.7759/cureus.99749

**Published:** 2025-12-21

**Authors:** Khalid Abi El Aala, Abdessamad Lalaoui, Ghizlane Kassal, Fatiha Bennaoui, Nadia El Idrissi Slitine, Fadl Mrabih Rabou Maoulainine

**Affiliations:** 1 Neonatal Intensive Care Unit, Mother and Child Hospital, Mohammed VI University Hospital Center, Marrakech, MAR; 2 Research Center for Childhood, Health and Sustainable Development, Cadi Ayyad University, Faculty of Medicine and Pharmacy of Marrakesh (FMPM), Marrakech, MAR

**Keywords:** case report, fetal hypoxia, hematoma, neonatal outcome, newborn, perinatal care, term infant, umbilical cord, umbilical cord pathology, wharton’s jelly

## Abstract

Umbilical cord hematoma is a rare but potentially severe condition associated with fetal distress and perinatal morbidity, and most cases are diagnosed retrospectively at birth. Early recognition is important due to the risk of fetal hypoxia. We report the case of a full-term female newborn (39 weeks of gestation, birth weight: 3,400 g) born vaginally without complications. The pregnancy was poorly monitored, but without known maternal risk factors. The APGAR score was 9 at the first minute. Immediate clinical examination revealed a 5 × 1.5 cm hematoma located at the fetal insertion of the umbilical cord, with marked dark red to black discoloration. The newborn showed no respiratory distress, hemodynamic instability, or signs of acute blood loss. Hemoglobin and bilirubin levels were within normal limits. Clinical monitoring remained uneventful, and the appearance of the cord improved significantly within 48 hours. This case illustrates a rare but often benign presentation of umbilical cord hematoma when fetal circulation is not compromised. Early postnatal evaluation and close monitoring are essential to exclude complications and ensure a favorable outcome.

## Introduction

Umbilical cord hematoma is an uncommon event defined as bleeding into Wharton’s jelly from ruptured umbilical vessels [1,2]. The estimated incidence ranges between 1 in 5,500 and 1 in 11,000 births [3]. The condition is clinically significant because it can compromise fetal blood flow, potentially resulting in hypoxia, stillbirth, or neonatal morbidity. Available data suggest that approximately 50% of reported umbilical cord hematomas are associated with fetal distress or adverse perinatal outcomes, underscoring the potentially serious nature of this condition [1,2,4]. However, some cases remain clinically silent and are discovered incidentally after birth [5]. This rarity supports the relevance of reporting individual cases to improve clinical awareness and management.

We report a case of spontaneous umbilical cord hematoma located at the fetal insertion in a full-term newborn with normal postnatal adaptation and favorable evolution.

## Case presentation

A full-term female newborn was delivered at 39 weeks of gestation to a 35-year-old mother with no significant medical history, specifically, no signs of local infection or personal or family history of coagulation disorders. The pregnancy was poorly monitored, characterized by only one prenatal visit. The requested initial assessments included a complete blood count, Rh factor testing, and fasting blood glucose. Crucially, no serological screening or prenatal ultrasound was performed, and no iron or vitamin supplements were administered. Labor progressed spontaneously, and fetal heart rate monitoring showed no anomalies. The total duration of labor was not precisely documented but was considered uncomplicated and non-instrumental.

**Figure 1:**
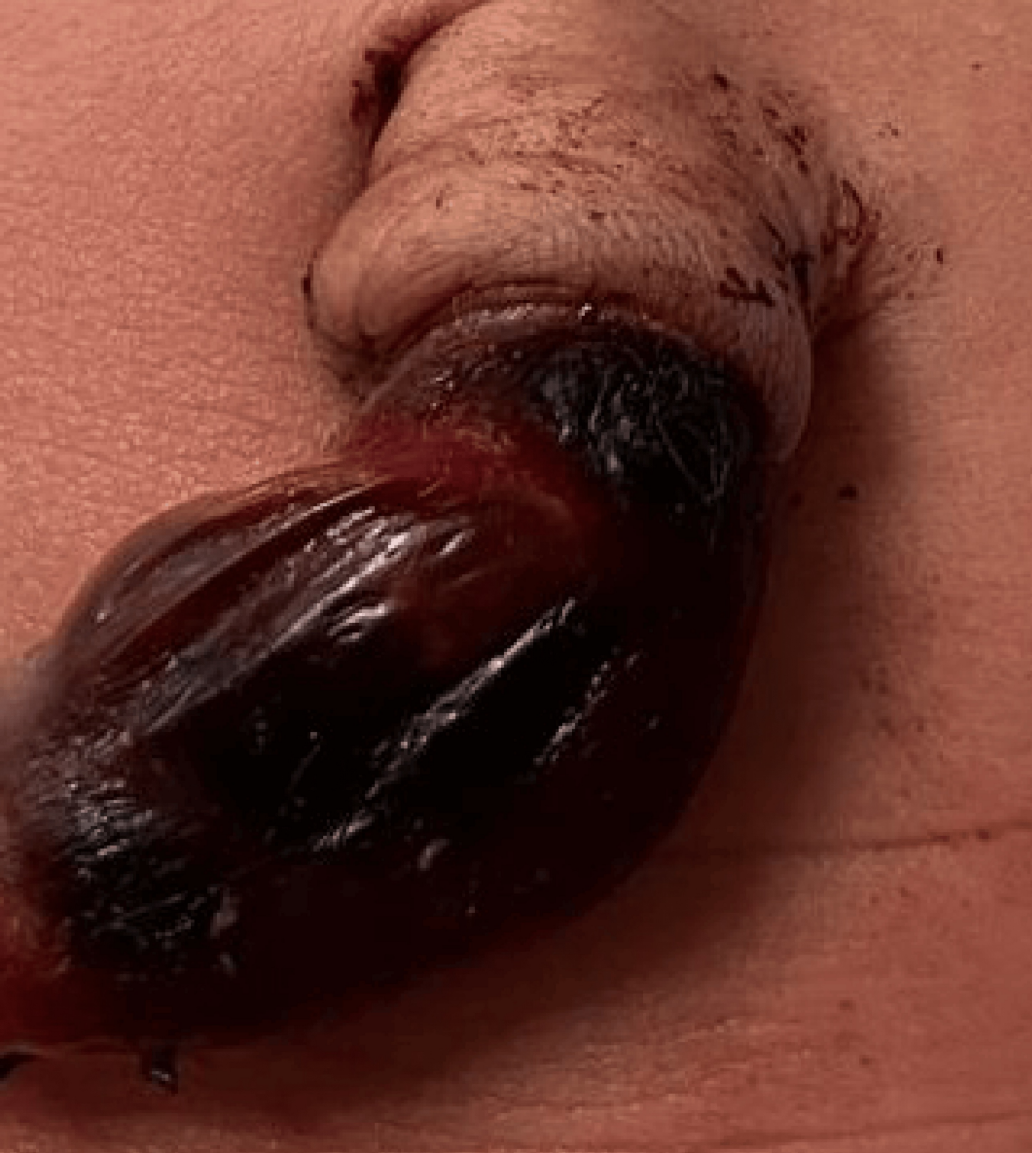
Umbilical cord hematoma measuring approximately 5 × 1.5 cm located at the fetal insertion, showing dark red to black discoloration consistent with hemorrhage into Wharton’s jelly

The newborn was delivered vaginally with an APGAR score of 9 at the first minute and a birth weight of 3,400 g. The umbilical cord was clamped immediately upon delivery due to the presence of the unexpected hematoma. Initial examination revealed a 5 × 1.5 cm umbilical cord hematoma located at the fetal insertion, characterized by a tense, dark red to black discoloration of the cord segment (Figure 1). No malformations or dysmorphic features were noted. Cardiovascular, respiratory, abdominal, and neurological examinations were normal. The newborn exhibited no respiratory distress and no hemodynamic compromise and required no resuscitation.

Laboratory evaluation showed normal hemoglobin and bilirubin levels. An immediate coagulation workup was performed, including prothrombin time (PT), activated partial thromboplastin time (aPTT), and platelet count, all of which were within the normal reference ranges for a term neonate. Clinical monitoring during the first 48 hours remained uneventful, with progressive improvement of the cord appearance.

## Discussion

Umbilical cord hematoma is an uncommon but potentially life-threatening condition resulting from the rupture of umbilical vessels, most frequently the umbilical vein, causing extravasation of blood into Wharton’s jelly [3,6]. This accumulation may rapidly increase cord diameter, compromise blood flow, and generate significant fetal distress if the hemorrhage is extensive. Although the exact incidence remains difficult to ascertain due to underreporting and variable diagnostic criteria, it is generally considered a rare event. Several risk factors have been postulated, including mechanical forces such as traction during labor, compression injuries, true knots, cord cysts, velamentous insertion, local infections, and congenital or acquired coagulation disorders [4,6]. Despite these associations, many cases occur in the absence of identifiable predisposing factors, thus remaining idiopathic [5].

The literature underscores that outcomes in umbilical cord hematoma depend largely on the timing and extent of vascular injury. Although most prenatally detected umbilical cord hematomas reported in the literature are associated with progressive enlargement and fetal compromise, rare cases have described limited or stable hematomas without significant hemodynamic impact. These cases, however, remain exceptional and are typically identified through close antenatal surveillance [1]. Prenatal, slowly developing hematomas can exert prolonged pressure on umbilical vessels, resulting in chronic fetal hypoxia, growth restriction, or even intrauterine demise. In contrast, acute hematomas occurring intrapartum tend to produce less severe consequences, provided that delivery is timely and the infant is promptly evaluated. Recent case series emphasize that early recognition, even when the diagnosis is made postnatally, and systematic neonatal assessment are essential to reducing morbidity. In spite of being potentially fatal, if detected in an asymptomatic newborn, it is associated with a good prognosis [7].

In the present case, the absence of documented antenatal Doppler ultrasound, together with normal fetal growth at birth, reassuring fetal heart rate monitoring during labor, and excellent neonatal adaptation, supports the likelihood of an acute event occurring late in gestation or during labor. Had the hematoma developed earlier in pregnancy, it would more likely have manifested as fetal growth restriction, abnormal Doppler findings, or other signs of chronic fetal compromise. The benign neonatal course observed is consistent with several previously reported cases in which acute hematomas formed intrapartum, thereby limiting the duration of fetoplacental compromise and improving prognosis [8,9].

From a clinical standpoint, careful postnatal monitoring is required to identify potential complications such as neonatal anemia, ongoing bleeding, hyperbilirubinemia, or hemodynamic instability. Although our patient remained clinically stable, this condition warrants heightened vigilance in the immediate postpartum period, including hemoglobin assessment, evaluation of perfusion, and surveillance for jaundice. The finding of a normal coagulation panel (PT, aPTT, and platelet count) in our patient is critical, as it effectively rules out a primary coagulation disorder as the cause of the umbilical cord hematoma, supporting a purely traumatic or mechanical etiology. In cases with significant hematoma size or suspected fetal compromise, additional investigations such as cord blood gas analysis, ultrasound assessment of residual hematoma, or serial hematological monitoring may be indicated.

Overall, our report reinforces that while umbilical cord hematoma can be associated with significant perinatal morbidity [10], the prognosis may be favorable when the event is acute, the neonate exhibits reassuring postnatal adaptation, and appropriate monitoring is ensured. Raising clinician awareness of this rare condition remains essential to facilitate early detection and optimal management.

## Conclusions

Umbilical cord hematoma is a rare but potentially serious condition. This case highlights that when detected postnatally without signs of fetal compromise, its evolution may be benign. Early neonatal assessment and follow-up remain essential to ensure a favorable outcome.

## References

[1] Scutiero G, Bernardi G, Iannone P, Nappi L, Morano D, Greco P (2018). Umbilical cord hematoma: a case report and review of the literature. Obstet Gynecol Int.

[2] Rabetsimamanga LZ, Rabarikoto HF, Rekoronirina EB, Andrianampanalinarivo HR (2018). Spontaneous umbilical cord hematoma causing still birth: a case report in Madagascar. Int J Reprod Contracept Obstet Gynecol.

[3] Benirschke K, Burton GJ, Baergen RN (2012). Pathology of the Human Placenta. Pathology of the Human Placenta.

[4] Olaya-C M, Bernal JE (2015). Clinical associations to abnormal umbilical cord length in Latin American newborns. J Neonatal Perinatal Med.

[5] de Laat MW, van der Meij JJ, Visser GH, Franx A, Nikkels PG (2007). Hypercoiling of the umbilical cord and placental maturation defect: associated pathology?. Pediatr Dev Pathol.

[6] Baergen RN (2013). Umbilical cord pathology. Surg Pathol Clin.

[7] Ferreira AC, Soares IP, Sousa S, Ferreira P, Ramos D (2023). Umbilical cord hematoma - a successful case of a non-frequent acute fetal distress etiology. Pregnancy Child Birth.

[8] Khatiwada P, Alsabri M, Wiredu S, Kusum V, Kiran V (2021). Spontaneous umbilical cord hematoma. Cureus.

[9] Mota F, Oliveira N, Fonseca M, Mimoso G (2019). Spontaneous umbilical cord haematoma. BMJ Case Rep.

[10] Sizun J, Soupre D, Broussine L (1995). [Spontaneous umbilical cord hematoma, a rare cause of acute fetal distress]. Arch Pediatr.

